# Prevalence of Anemia Among the Elderly in India: Evidence From a Systematic Review and Meta-Analysis of Cross-Sectional Studies

**DOI:** 10.7759/cureus.42333

**Published:** 2023-07-23

**Authors:** Roy A Daniel, Farhad Ahamed, Suprakash Mandal, Vignesh Lognathan, Tandra Ghosh, Gomathi Ramaswamy

**Affiliations:** 1 Centre for Community Medicine, All India Institute of Medical Sciences, New Delhi, New Delhi, IND; 2 Department of Community and Family Medicine, All India Institute of Medical Sciences, Kalyani, Saguna, IND; 3 Department of Community Medicine, Jawaharlal Institute of Postgraduate Medical Education & Research, Pondicherry, IND; 4 Department of Physiology, All India Institute of Medical Sciences, Kalyani, Saguna, IND; 5 Department of Preventive and Social Medicine, All India Institute of Medical Sciences, Bibinagar, Bibinagar, IND

**Keywords:** who criteria for adult anemia, hemocue, sahli’s method, pooled prevalence of anemia, old age home, hemoglobin, india, meta-analysis, elderly, anemia

## Abstract

Anemia is a leading cause of increased morbidity and mortality among the elderly population. In spite of numerous interventions and strategies rolled out to tackle the growing burden of anemia, lesser importance is being given to this age group. There is a lack of data on the national level burden of anemia among elderly persons (≥ 60 years) in India. We aimed at estimating the prevalence of anemia among elderly persons (≥ 60 years) in India by conducting a systematic review and meta-analysis. We searched PubMed, Embase, Cochrane Library, Google Scholar, and IndMed, and included cross-sectional studies reporting data on the prevalence of anemia among elderly persons in India and used random effects model to estimate pooled point prevalence with 95% confidence interval (CI), To explore the heterogeneity further, we did sub-group analyses based on zonal divisions of India (region), rural or urban, study setting, method of hemoglobin estimation and sampling strategy. Out of 22 studies, one study was of high quality of bias, 11 of moderate, and 10 were of low quality of bias. The pooled estimate of anemia was 68.3% (95%CI: 60.7 to 75.9), I^2^ = 99.0%, and Q=2079.2 (p-value <0.001). The pooled prevalence of anemia among the elderly in India was found to be high and necessary actions need to be taken at the policy level to achieve “active and healthy ageing”.

## Introduction and background

Anemia is a common, multi-factorial condition affecting all age groups and after the age of 50 years, the prevalence rises [1,2]. The World Health Organization (WHO) defines anemia as hemoglobin less than 13g/dl and 12g/dl for males and females, respectively [1]. Elderly age groups (≥ 60 years) are most vulnerable to anemia since age-related changes in bone marrow, poor absorption, and other metabolic changes lead to inadequate synthesis of hematopoietic blood cells and further reduction in hemoglobin [3,4]. In addition, according to a global estimate, 80% of all older people would reside in low- and middle-income nations by the year 2050 [5]. Elderly anemia has been independently linked to deteriorated physical and cognitive abilities [6], increased dementia [7], and increased risk of falls, morbidity, and mortality [8]. It is also an independent predictor of poor health outcomes in elderly patients and is a risk factor for cardiovascular and neurological events [9,10]. Altogether, anemia among the elderly population grossly affects the health-related quality of life [11]. Moreover, many anemia symptoms, such as weakness, exhaustion, and shortness of breath, are frequently misunderstood as common occurrences in elderly people. Therefore, early detection of anemia in the elderly is necessary to prevent delay in diagnosis of potentially other treatable conditions. Anemia in the elderly is little understood clinically, and there are no evidence-based management recommendations, particularly from a public health standpoint.

As per a WHO report, the global prevalence of anemia among the elderly population was 23.9% affecting 164 million individuals [12]. The burden of anemia estimated from various studies across the globe ranged from 8.8% in Italy to 45.5% in India [13,14]. In India, there is a lack of national-level estimates on the prevalence of anemia among the elderly, which will be useful to assess the burden of anemia in the elderly in India. The National Family Health Survey has estimated hemoglobin levels only till 49 years of age [15]. This estimation is essential to plan appropriate and precise strategies to avoid the precarious consequences of anemia. Hence, we conducted a systematic review and meta-analysis to provide a holistic estimate of the prevalence of anemia among elderly persons (≥ 60 years) in India.

## Review

Methods

Literature Search Strategy

Between October 2022 and December 2022, a thorough literature search was conducted using the PubMed/MEDLINE, Embase, IndMed, Cochrane Library, and Google Scholar databases to find studies that had been published up to December 31, 2022. Search terms relating to the results were combined with Medical Subject Headings (MeSH) and free text words (prevalence, burden, anemia, hemoglobin, elderly, India). We followed the Preferred Reporting Items for Systematic Reviews and Meta-Analyses (PRISMA) guidelines and Meta-analysis of Observational Studies in Epidemiology (MOOSE) standards [16,17]. The protocol for the review was registered in the International Prospective Register of Systematic Reviews or PROSPERO (number: CRD42020218195).

Selection Criteria

An initial screening of selected titles and abstracts was done, followed by a full-text review. Only observational studies that reported anemia prevalence and met the inclusion criteria were considered. Inclusion criteria were as follows: (i) cross-sectional study design, (ii) conducted among elderly persons (≥ 60 years) on the prevalence of anemia, (iii) conducted in either a population/community-based, hospital-based, or any specific setting like old age home, etc. in India, and (iv) sufficient data was available in the article to extract the numerator and denominator for the prevalence of anemia in the age group ≥ 60 years. Exclusion criteria were as follows: (i) studies assessing anemia in the elderly with specific conditions like anemia with chronic kidney disease, etc., and (ii) letters, abstracts, conference proceedings, reviews, and studies not conducted on humans.

Study Selection

All the titles of the records that were retrieved from the databases were reviewed by two independent reviewers (SM and VL), who then looked into the abstracts of pertinent titles. If an abstract met the criteria, it was chosen. Any selection-related disputes were discussed with RAD in order to be resolved. After confirming the most recent and comprehensive version, all duplicates were eliminated. For the chosen abstracts, full-text studies were located. We looked through the reference lists of the studies to locate additional sources. To make sure they met the inclusion criteria, additional evaluations of the retrieved full-text papers were performed.

Data Extraction

We created an Excel 2013 data collecting form (Microsoft Corporation, Redmond, Washington, United States) to extract and insert the pertinent data fields from the chosen full-text studies. The data collection sheet contained information about the authors, the year the article was published, the study's setting (rural or urban), the strategy for sampling, the sample size, the method for estimating hemoglobin, the classification criteria for anemia, and the reported prevalence of anemia. The quality of the research included in this review was evaluated using the New Castle Ottawa Scale (NOS) [18], adapted for cross-sectional studies [19] Studies with a score of 8 or higher were deemed to be of high quality, those with a score of 4-7 were deemed to be of moderate quality, and those with a score of 3 were deemed to be of low quality.

Statistical Analysis

The prevalence of anemia served as the outcome metric. The reported prevalence and the sample size for each study were used to compute the standard error (SE) of the prevalence using the formula "square root of p x (1-p)/n." The accuracy of the summary estimations was evaluated using a 95% confidence interval (CI). The random effects model, weighted by the inverse of variance, was used to perform the meta-analysis via package metan [20] in STATA Release 13 (2013; StataCorp LP, College Station, Texas, United States) [21]. Heterogeneity was assessed using Cochrane's Q statistic test and the I^2^ statistic (percentage of residual variance ascribed to heterogeneity). The pooled prevalence and its 95%CIs were presented in the pooled analysis. By visually examining the funnel plot, publication bias, and the small-study impact were both evaluated by funnel plot and Egger’s test. The method of hemoglobin measurement, sampling method, rural vs urban study setting, and zonal divisions of India (region) [15] were used in the subgroup analysis. Based on the study's quality, criteria, setting, and sample size, a sensitivity analysis was conducted. A test of interaction was also conducted to determine whether there was a difference in anemia prevalence between subgroups that were statistically significant.

Results

Study Selection

**Figure 1:**
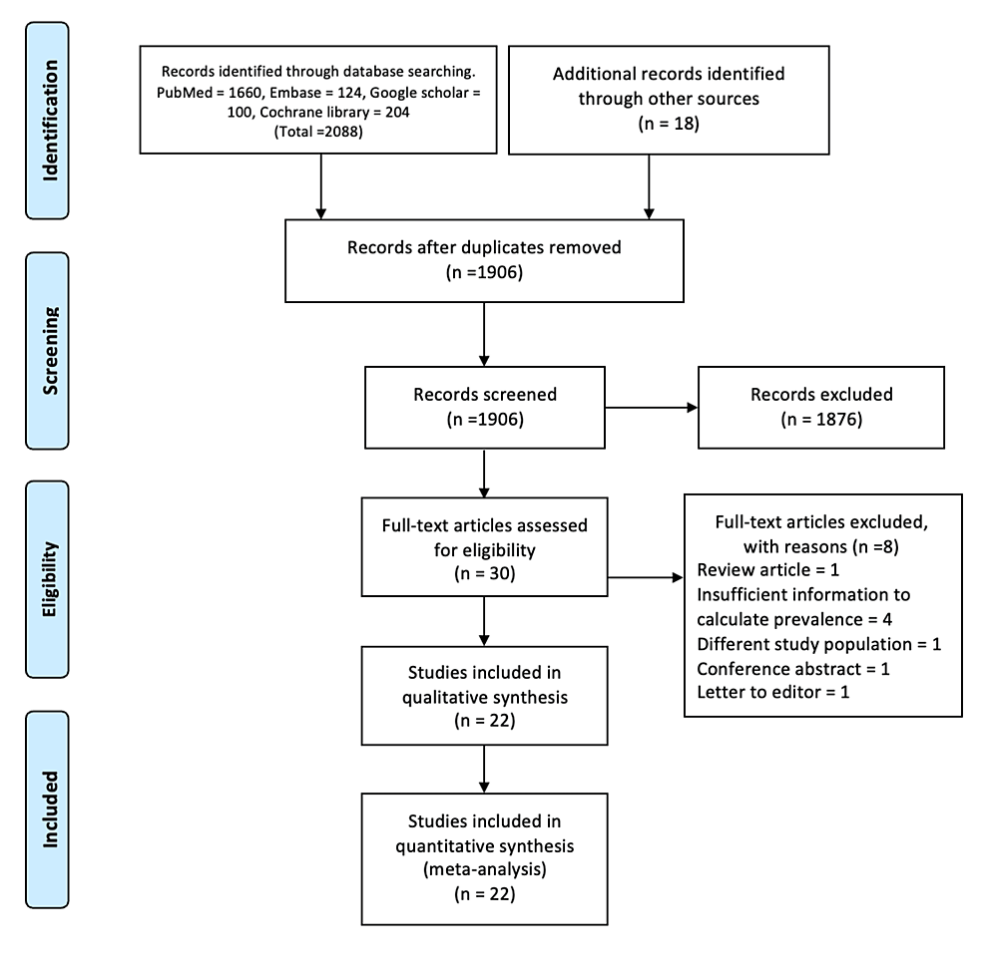
Flow of selection of studies for meta-analysis

Overall, 2106 studies were retrieved from the databases initially. After removing duplicates of studies, 1906 studies were screened and 1876 were excluded. A total of 30 eligible abstracts were screened by inclusion criteria, followed by a screening of the full text of the studies. Finally, 22 studies satisfied the inclusion criteria and were included in the meta-analysis (Figure 1).

Characteristics of Studies Included in the Meta-Analysis

We included a total of 8,501 individuals in the meta-analysis. The characteristics of studies included in the meta-analysis are shown in Table 1. Of the 22 studies, the majority of them used a simple random sampling method followed by cluster random sampling, and multistage random sampling. Most of the studies were in an urban locality. The majority of the studies were community-based; two studies were hospital-based and two were old-age-home based. Among studies that had mentioned the method that was used to estimate hemoglobin, the cyanmethemoglobin method was the most common followed by HemoCue System (Danaher Corporation, Washington, D.C., United States), and Sahli’s method. WHO’s hemoglobin cut-off for anemia was used by all the studies except Agarwal et al. [22] and Swami et al. [23], who did not mention the criteria that they used. Singh et al. [24], Gonmei et al. [25,26], and Maninder et al. [27] conducted studies only among elderly females. All the studies were cross-sectional studies and none of them were multisite or nationally representative.

**Table 1:** The characteristics of the studies included in the systematic review and meta-analysis ^*^WHO: World Health Organization; ^†^Not available

S.No.	Author	Study area in India	Study setting	Sample size (≥ 60 years)	Sampling strategy	Hb estimation method	Diagnostic criteria	Prevalence of anemia (%)
1	Agrawal et al. [22], 2011	Maharashtra	Rural, Community-based	214	Random sampling method	Sahli's method	NA	62.6
2	Swami et al. [23], 2002	Chandigarh	Urban & Rural, Community-based	362	Stratified random technique	Sahli's method	NA†	68.2
3	Singh et al. [24], 2018	New Delhi	Urban, Community-based	512	Random sampling method	HemoCue	WHO	79.9
4	Gonmei et al. [25], 2017	New Delhi	Urban, Community-based	60	NA	Cyanmethemoglobin method	WHO	66.7
5	Gonmei et al. [26], 2018	New Delhi	Urban, Community-based	116	NA	Cyanmethemoglobin method	WHO	57.7
6	Kaur et al. [27], 2009	Haryana	Urban & Rural, Community-based	200	Purposive sampling	Cyanmethemoglobin method	WHO	93.5
7	Vadakattu et al. [28], 2019	Telangana	Urban, Community-based	282	Random sampling method	Cyanmethemoglobin method	WHO	20.6
8	Sudarshan et al. [29], 2016	Puducherry	Rural, Community-based	360	Random sampling method	NA	WHO	96
9	Bharati et al. [30], 2011	Puducherry	Urban & Rural, Community-based	214	NA	NA	WHO	86
10	Shrivastava et al. [31], 2013	Karnataka	Urban, Hospital-based	654	NA	NA	WHO	68.5
11	Maiti et al. [32], 2013	West Bengal	Rural, Community-based	544	NA	HemoCue	WHO	89.5
12	Punia et al. [33], 2015	Haryana	Rural, Community-based	982	Random sampling method	Cyanmethemoglobin method	WHO	90
13	Paul et al. [34], 2015	Tamil Nadu	Rural, Community-based	340	Multistage random sampling	NA	WHO	38.2
14	Agarwalla et al [35], 2016	Assam	Rural, Community-based	330	Cluster sampling	Sahli's method	WHO	45.5
15	Soni et al. [36] 2016	Maharashtra	Urban, Hospital-based	550	NA	NA	WHO	67.1
16	Vijayakumar et al. [37], 2018	Puducherry	Rural, Community-based	250	Population proportionate to size	HemoCue	WHO	80.8
17	Pathania et al. [38], 2019	New Delhi	Urban, Old-age home-based	334	Cluster random sampling	HemoCue	WHO	68.7
18	Renjini et al. [39]. 2019	Kerala	Urban, Old-age home-based	104	NA	HemoCue	WHO	76
19	Kant et al. [40], 2019	Haryana	Rural, Community-based	175	Multistage random sampling	HemoCue	WHO	46.8
20	Lamba et al. [41], 2019	Uttar Pradesh	Urban, Community-based	395	Simple Random sampling	Haemo Check Rapid Diagnostic Kit	WHO	49.6
21	Gupta et al. [42], 2020	Uttarakhand	Rural, Community-based	958	Population proportionate to size	Cyanmethemoglobin method	WHO*	92.1
22	Retnakumar et al. [43], 2020	Kerala	Urban, Community-based	165	Purposive	HemoCue	WHO	60.6

Risk of Bias Assessment

Out of 22 studies, one study was of high quality, 11 studies were of moderate quality, and 10 studies were of low quality of bias (Table 2). Among the included studies, 14 studies used validated instruments for hemoglobin estimation, 13 studies used random sampling strategies to select participants, and five studies used appropriate and complete statistical tests to analyze and present the findings.

**Table 2:** Risk of bias assessment for all the selected studies

S.No.	Author	Selection	Comparability	Outcome	Quality score
1	Agrawal et al. [22], 2011	4	2	1	7
2	Swami et al. [23], 2002	5	1	1	7
3	Singh et al. [24], 2018	4	0	0	4
4	Gonmei et al. [25], 2017	5	0	0	5
5	Gonmei et al. [26], 2018	2	0	0	2
6	Kaur et al. [27], 2009	5	1	0	6
7	Vadakattu et al. [28], 2019	3	0	0	3
8	Sudarshan et al. [29], 2016	3	0	0	3
9	Bharati et al. [30], 2011	4	2	1	7
10	Shrivastava et al. [31], 2013	2	0	0	2
11	Maiti et al. [32], 2013	3	0	0	3
12	Punia et al. [33], 2015	5	2	1	8
13	Paul et al. [34], 2015	2	1	1	4
14	Agarwalla et al. [35], 2016	0	0	0	0
15	Soni et al. [36], 2016	0	0	0	0
16	Vijayakumar et al. [37], 2018	2	0	0	2
17	Pathania et al. [38], 2019	5	2	0	7
18	Renjini et al. [39]. 2019	2	0	0	2
19	Kant et al. [40], 2019	2	0	0	2
20	Lamba et al. [41], 2019	0	2	0	2
21	Gupta et al. [42], 2020	3	0	1	4
22	Retnakumar et al. [43], 2020	4	2	1	7

Prevalence of Anemia Among the Elderly in India

Prevalence of anemia among the elderly (n=22 studies) ranged from 20.6% in a study conducted by Vadakattu et al. [28] in an urban locality of Telangana, to 96% in a study conducted by Sudarshan et al. [29] in a rural locality of Puducherry.

**Figure 2:**
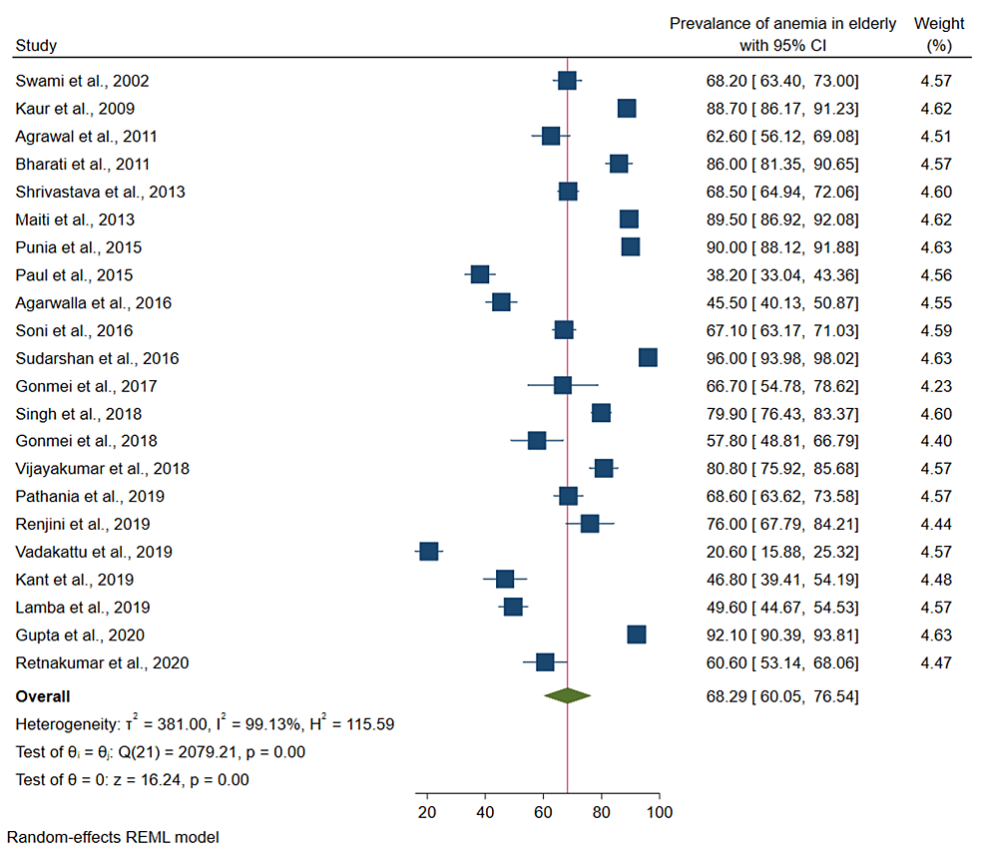
Forest plot of the meta-analysis for the prevalence of anemia among elderly Sources: References [22-43]

Random effects pooled estimate: The random effects pooled estimate for the prevalence of anemia among the elderly in India was 68.3% (95%CI: 60.7-75.9) (Figure 2). There was significant heterogeneity between the studies. Heterogeneity test showed I^2^=99.0%, Q = 2079.2, and p-value <0.001.

Sub-Group Analysis

**Figure 3:**
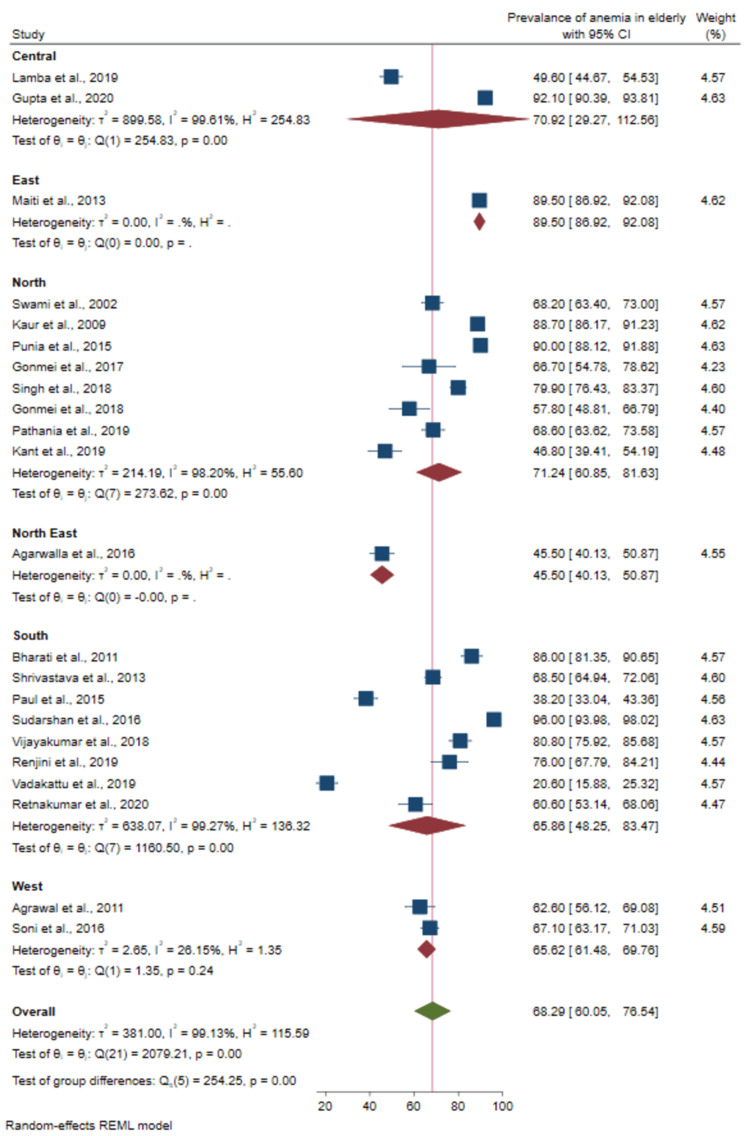
Forest plot of the meta-analysis for the prevalence of anemia among elderly based on geographical region Sources: References [22-26, 28-43]

Prevalence of anemia based on geographical region: Based on the zonal divisions of India, studies conducted in the west region demonstrated a mild heterogeneity (I^2^=26.1%, p-value = 0.245). There was a significant difference in prevalence in the studies categorized based on geographical regions in India as shown in Figure 3 (p-value = <0.001). The prevalence of anemia among the elderly among various sub-groups is shown in Table 3.

**Table 3:** Prevalence of anemia among elderly persons by sub-groups

Sub-groups	No. of studies	No. of participants	Prevalence (%) with 95% CI	Heterogeneity tests		p-value (sub-group difference)
				I^2^%	Q	
Geographical region						
Central	2	1353	70.9 (29.3- 112.6)	99.6	254.8	<0.001
North	8	3141	71.4 (60.9-81.6)	98.2	273.6	
West	2	764	65.6 (61.5-69.8)	26.1	1.4	
South	8	2369	65.9 (48.3-83.5)	99.3	1160.5	
East	1	544	89.5 (86.9-92.1)	-	-	
North East	1	330	45.5 (40.1-50.9)	-	-	
Hb estimation method						
Cyanmethemoglobin	6	2598	69.4 (47.1-91.7)	99.7	855.9	<0.001
Hemocue rapid diagnostic kit	1	395	49.6 (44.7-54.6)	-	-	
HemoCue	7	2084	71.9 (61.4-82.6)	97.2	176.9	
Sahli’s method	3	906	58.8 (45.3-72.2)	94.4	39.5	
Locality						
Rural	9	4153	71.4 (56.4-86.5)	99.6	869.9	0.06
Urban	10	3172	61.5 (50.9-72.1)	97.7	459.3	
Urban and Rural	3	1176	81.1 (68.5-93.6)	96.7	55.3	
Study setting						
Community	18	6859	67.8 (57.8-77.9)	99.3	1932.7	<0.001
Old-age home	2	438	71.6 (64.5-78.6)	56.2	2.2	
Hospital	2	1204	67.9 (65.2-70.5)	0.0	0.2	
Sampling strategy						
Population proportionate to size	2	1208	92.1 (90.4-93.8)	-	-	<0.001
Cluster random sampling	2	664	57.1 (34.4-79.9)	97.4	38.6	
Random sampling	6	2745	69.9 (41.0-89.9)	99.6	909.5	
Purposive sampling	2	765	77.2 (45.0-109.5)	98.4	61.8	
Stratified random sampling	1	362	68.2 (63.4-73.0)	-	-	
Multistage random sampling	2	515	42.1 (33.7-50.5)	71.4	3.5	

**Figure 4:**
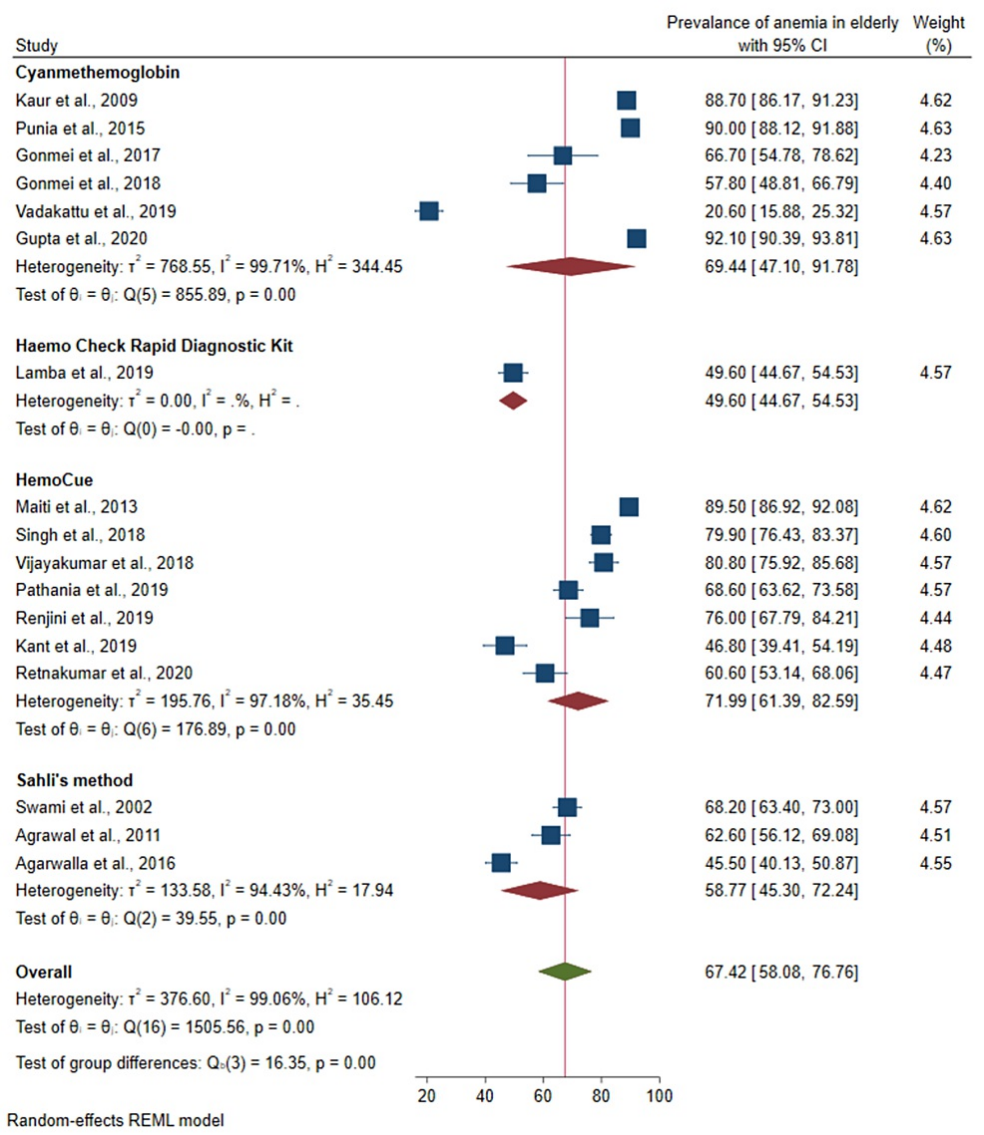
Forest plot of the meta-analysis for the prevalence of anemia among the elderly based on the hemoglobin estimation method Sources: References [22-28,32,33,35,37-43]

Prevalence of anemia based on hemoglobin estimation method: Out of the 22 studies, five studies did not mention the method that was used for hemoglobin estimation and so were excluded from the analysis. There was a significant difference in the prevalence between studies based on the hemoglobin estimation method (p-value < 0.001) as shown in Figure 4.

**Figure 5:**
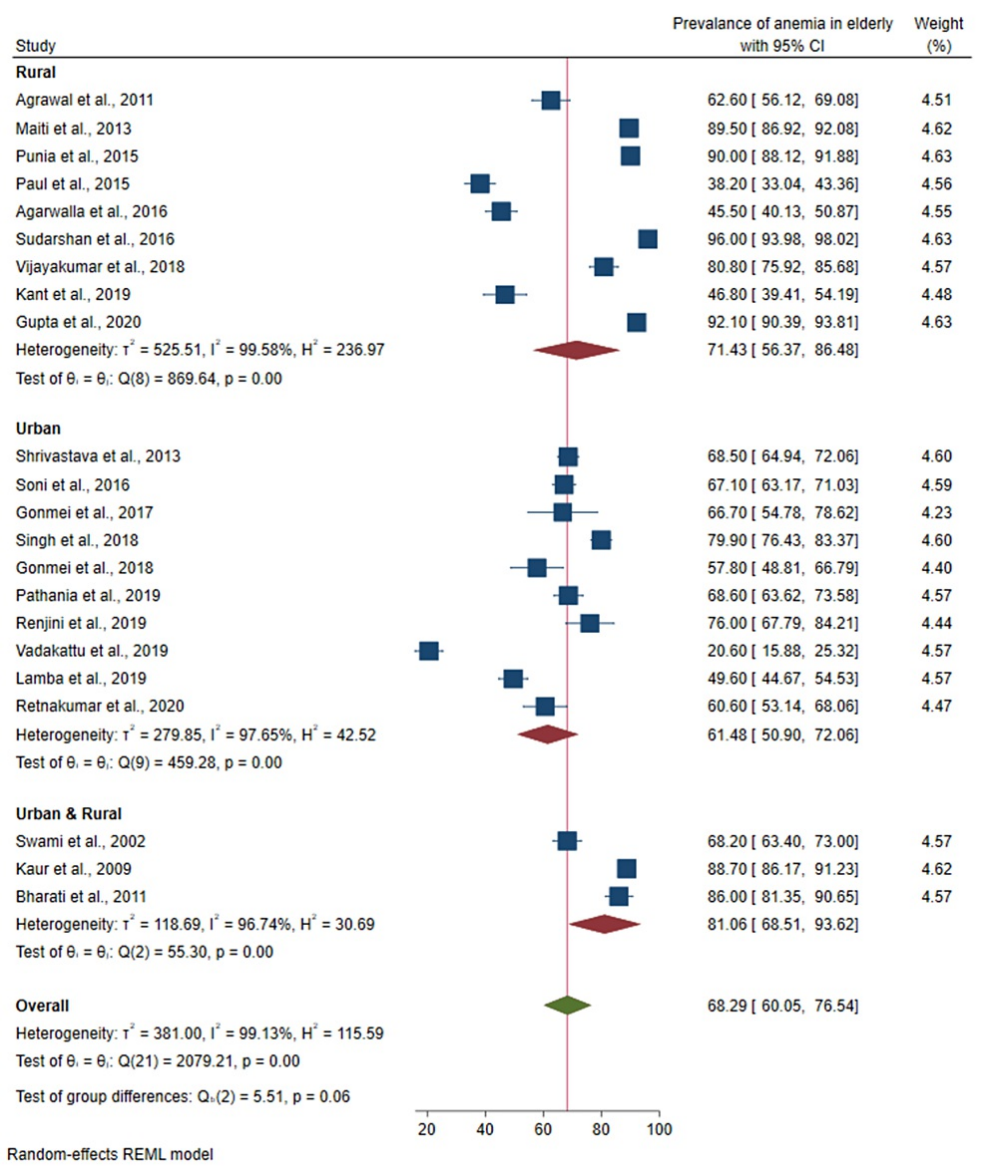
Forest plot of the meta-analysis for the prevalence of anemia among the elderly based on locality Sources: References [22-43]

Prevalence of anemia based on locality:Of the total of 22 studies, nine studies were conducted in rural areas, 10 from urban, and three from both urban and rural areas. There was no significant difference in the prevalence between studies based on the locality (p-value 0.06) as shown in Figure 5.

**Figure 6:**
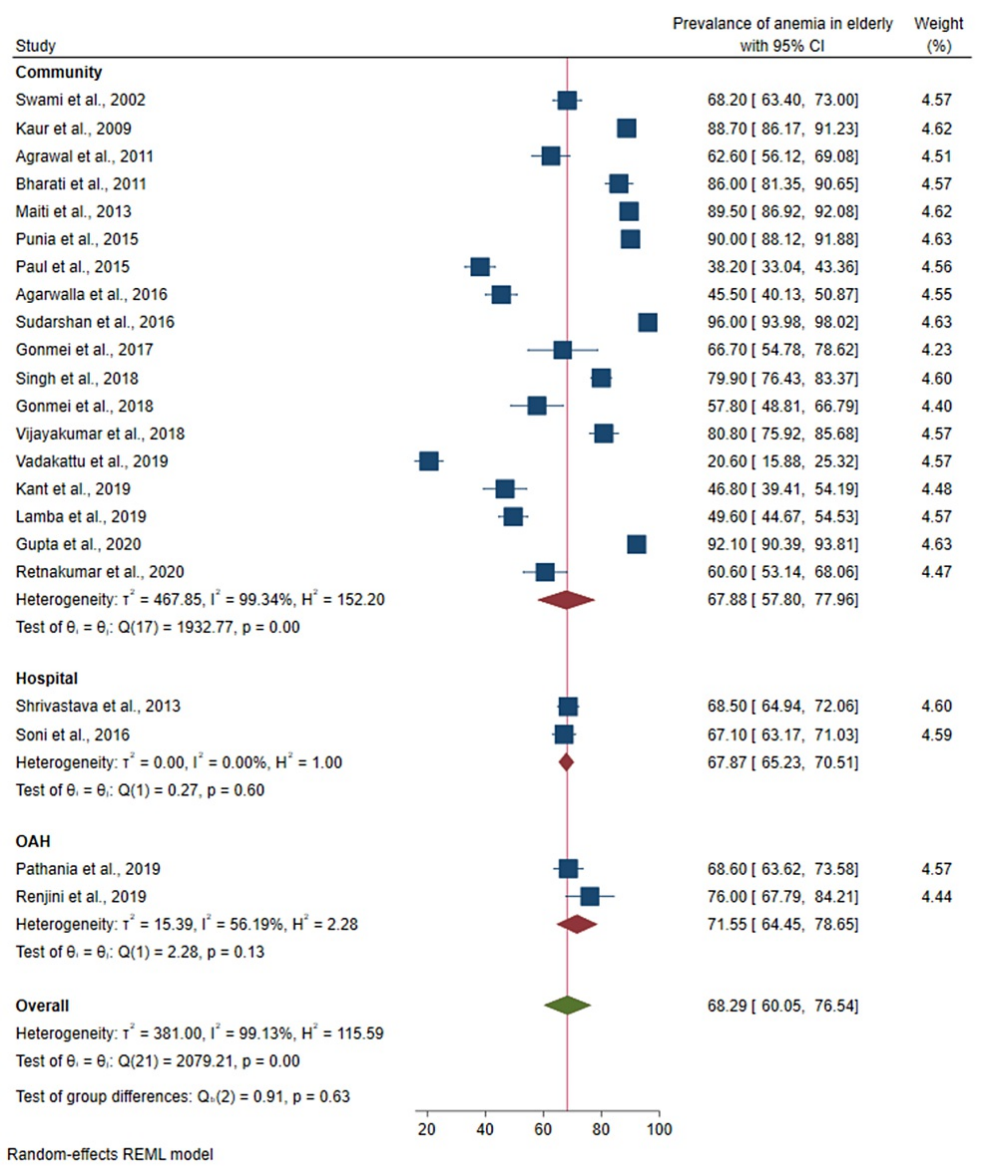
Forest plot of the meta-analysis for the prevalence of anemia among the elderly based on study settings Sources: References [22-43]

Prevalence of anemia based on study settings: Studies that were hospital-based demonstrated zero heterogeneity (I^2^ = 0, p-value = 0.600). There was a significant difference in the prevalence between studies based on study setting (p-value < 0.001) as shown in Figure 6.

**Figure 7:**
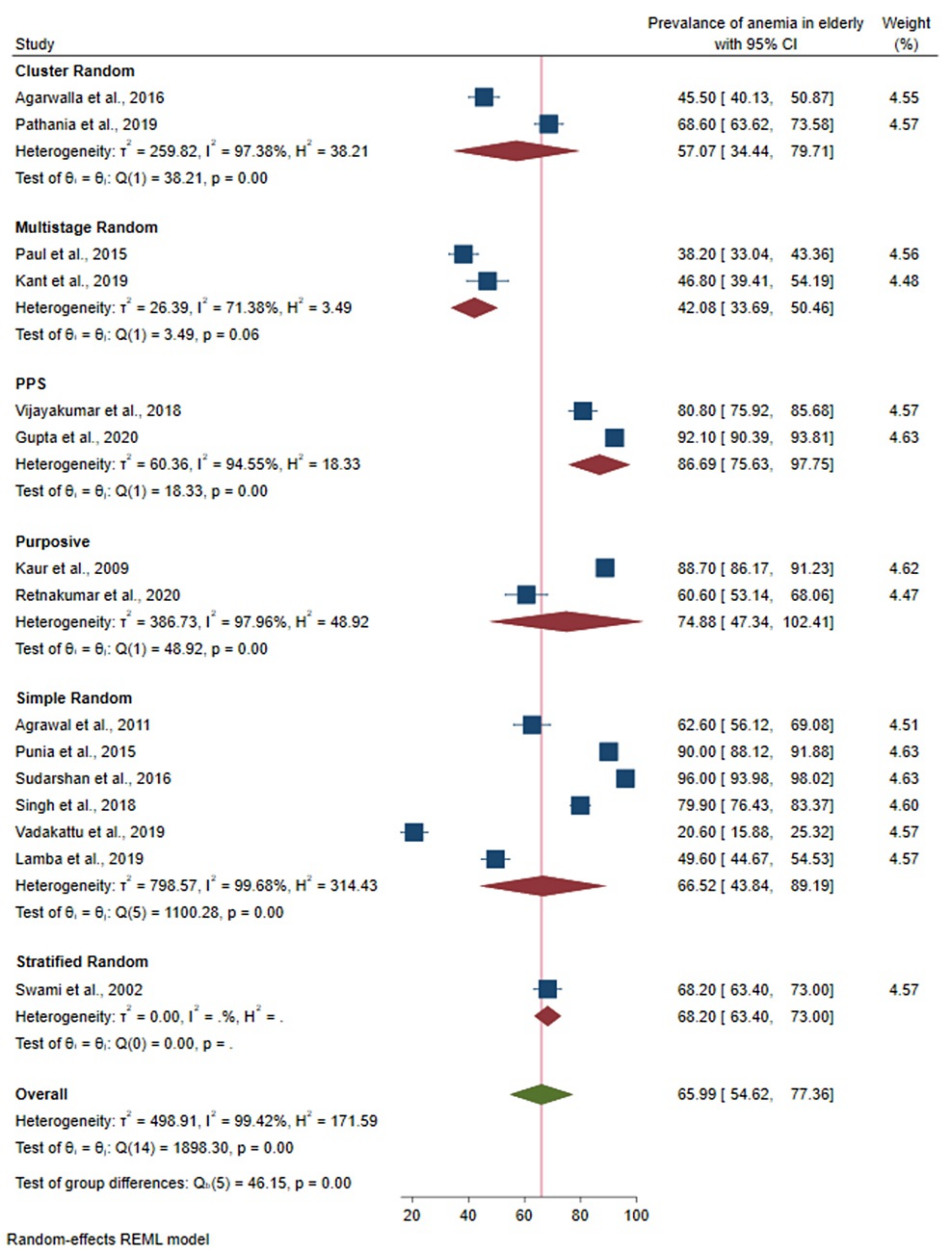
Forest plot of the meta-analysis for the prevalence of anemia among the elderly based on sampling design Sources: References [22-24,27-29,33-35,37,38,40-43]

Prevalence of anemia based on sampling design: Out of 22 studies, seven did not mention the sampling strategy. There was a significant difference in the prevalence between studies based on sampling strategy (p-value < 0.001) as shown in Figure 7.

Publication Bias

**Figure 8:**
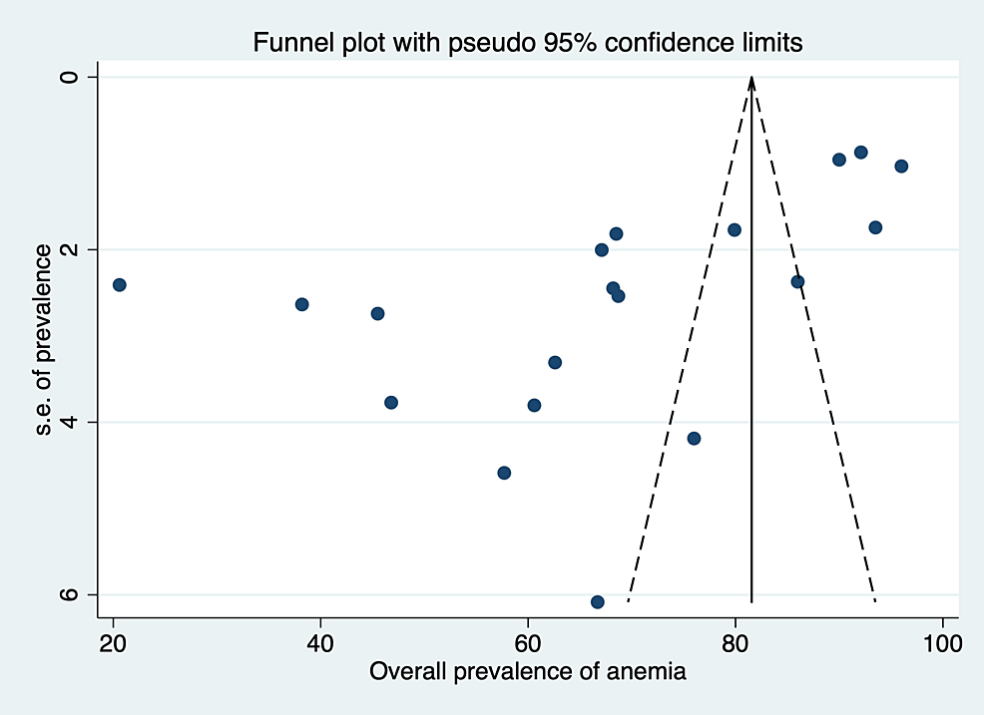
Funnel plot for publication bias

Funnel plot demonstrated asymmetry as shown in Figure 8. This was further confirmed by Egger’s test (p-value <0.01), implicating publication bias.

Sensitivity Analysis

Sensitivity analysis was performed by removing five studies that had a sample size of less than 200 and it showed only a 1% increase in prevalence from 68.3% (95%CI: 60.7-75.9) to 69.6% (95%CI: 56.6-82.7) as shown in Table 4. We re-ran the analysis for only those studies conducted in the community and the prevalence came out to be 67.8% (57.8-77.4%). The pooled estimate after removing two studies that did not mention detail on the cut-off criteria used to classify hemoglobin was 67.4% (58.1-76.8%). The pooled estimate after removing 10 low-quality studies showed a 1% increase in the prevalence (69.6% (56.6-82.7%)).

**Table 4:** Sensitivity analysis for the prevalence of anemia among the elderly

S. No	Sensitivity analysis	Prevalence of anemia with 95% CI	Heterogeneity tests		p-value
			I^2^%	Q	
1.	Removing studies that did not mention the cut-off	67.4 (58.1-76.8)	99.1	1505.6	<0.001
2.	Removing studies that was conducted in hospital and old-age home setting	67.8 (57.8-77.4)	99.3	1932.7	<0.001
3.	Removing studies that had a sample size of less than 200	61.3 (51.6-71.1)	84.6	28.2	<0.001
4.	Removing studies that were of low quality	69.6 (56.6-82.7)	99.1	1033.7	<0.001

Discussion

In our meta-analysis, the pooled prevalence of anemia in the elderly population was 68.3% (95%CI: 60.7-75.9). The pooled prevalence was almost six times higher than the prevalence of anemia estimated in the United States, where the overall prevalence of anemia among individuals aged more than 65 years was 10.6% [3]. A systematic review based on data from 45 studies done in developed countries, which included 85,409 individuals, reported the pooled prevalence of anemia as 17.0% in the elderly population [44]. It is important to mention here that earlier studies reported a much higher prevalence of anemia among individuals across all age groups living in developing countries like India than those living in developed countries [12]. In the absence of any national-level estimate of anemia prevalence specifically for the elderly age group, we compared our study findings with the NFHS-4 (2015-16) data and we observed that the pooled prevalence of anemia in the elderly was the highest among all other age groups in India. It is almost 18% higher than the anemia prevalence in pregnant women and women of reproductive age group [15].

We found that the prevalence of anemia was highest in the eastern, central, and northern states of India. North-eastern states of India had the least prevalence of anemia. A similar pattern of regional differences in the prevalence of anemia has been observed among adult males (15-54 years) and females (15-49 years) in the NFHS-4 survey [15]. Socio-economic status and dietary patterns were found to be associated with high anemia prevalence in younger age groups in selected Indian states [45]. However, one must evaluate the reasons for such regional differences in the prevalence of anemia in the elderly. Nonetheless, it is worth mentioning that in spite of regional variation, anemia prevalence in the elderly age range was extremely high across the board in all regions, which demands the immediate attention of decision-makers.

The prevalence of anemia also varied depending upon the method of anemia estimation employed in the individual studies. The highest prevalence of anemia was found in the studies that used the HemoCue method (71.9%; 95%CI: 61.4-82.6) followed by the cyanmethemoglobin method (69.4% (47.1%-91.7%)). Though the cyanmethemoglobin method is considered as the gold standard for the estimation of hemoglobin [46], the sensitivity and specificity of HemoCue are high and considered a very accurate method for the measurement of hemoglobin [47]. Studies that used Sahli’s method reported a relatively lower pooled estimate of anemia prevalence (58.8%; 95%CI: 45.3-72.2). Though Sahli’s method has a subjective component, it has been proved that in study settings, its validity was comparable to HemoCue [48].

The prevalence of anemia was marginally higher among studies conducted in rural areas (71.4%; 95%CI: 56.4-86.5) than in the studies from urban areas (61.5%; 95%CI: 50.9-72.1). This observation is almost universal in India across all age groups [49]. Better standard of living, income, diet, and treatment-seeking behavior might be the possible reasons behind such observation. However, in spite of this urban-rural variation, the prevalence of anemia was very high in both settings, which again should draw the attention of policymakers for immediate corrective action.

On sub-group analysis, we found that the prevalence of anemia based on the studies done in old age homes showed a higher prevalence of anemia (71.6%; 95%CI: 64.5-78.6) than those in the community (67.8%; 95%CI: 57.8-77.9) and hospital-based studies (67.9%; 95%CI: 65.2-70.5). The pooled prevalence of anemia between community-based studies and hospital-based studies was almost similar. Unlike our study, Gaskell et al. reported a higher prevalence of anemia among hospital-based studies than community-based studies [44]. The hospital-based studies included in our meta-analysis recruited individuals who were apparently not suffering from other chronic illnesses. Thus, the study population was almost representative of community-dwelling individuals. Moreover, studies that measured anemia associated with other chronic diseases were excluded from our study.

To summarize, the prevalence of anemia among the elderly is high as a total and also in various sub-groups. This high prevalence necessitates prompt inclusion of the elderly age group to determine the prevalence of anemia in this age group in national-level surveys. We also advocate the current national program for anemia to include the older age group [50]. We also conducted a sensitivity analysis to check for consistency in the prevalence based on the assumptions that might impact the prevalence of anemia. There was no significant difference in the prevalence of anemia in the sensitivity analysis. This shows the robustness of combining the articles.

However, we also want to suggest that the cut-off values for diagnosing anemia in the older age category be reviewed. According to a study, hemoglobin levels fall as people age, so determining the true burden of anemia in the elderly requires an age-adjusted cut-off for diagnosis [51]. The WHO criteria for adult anemia diagnosis were released in 1968, making them over five decades old and not yet updated. Researchers frequently apply the same cut-off for elderly adults because there are no specific criteria for identifying anemia in the elderly. Numerous studies have demonstrated that older individuals should not use the WHO criteria for defining anemia [52,53].

Anemia is a global public health problem and needs appropriate and timely intervention to promote health and prevent the consequences. Along with iron and folic acid, we may require multipronged intervention to overcome anemia in the elderly. As etiologies of anemia in the elderly differ from other age groups, proper classification of anemia needs to be established for a targeted approach. However, in 80% of cases of anemia in the elderly, the cause can be established [54]. None of the studies in our meta-analysis included an etiological classification of anemia. Thus, the etiologies of anemia in the elderly at the national level have to be assessed in community settings so that proper preventive and curative measures can be adopted at the program level. It is important to mention here that studies in developed countries reported that anemia in the elderly can be broadly classified into two categories: nutritional and non-nutritional (genetic disorders, environmental conditions, infections, inflammations, gastrointestinal abnormality, etc.), and each category contributes almost in equal proportion [3].

There was high heterogeneity across the selected studies in this meta-analysis. The various reasons for this high heterogeneity might be due to the varied sampling strategies employed in the studies, methods used to estimate hemoglobin, and study settings. To explore the heterogeneity, we did a sub-group analysis and found that studies conducted in the western region of India and studies conducted in hospitals showed small heterogeneity. However, the prevalence of anemia across various sub-groups did not differ much and was universally high. We tried to identify the reason for a large heterogeneity through sub-group analysis and sensitivity analysis. However, the I^2^ statistic was not lowered for any variable which was studied for heterogeneity. The most probable reason for this large amount of heterogeneity could be differences in methodology rather than statistical or biological factors; however, those factors were not objectively classified and studied in this study.

Given the high burden of anemia among the elderly, they should be added as one of the age groups for inclusion in the Anemia Mukt Bharat program of the Government of India. National surveys like NFHS should include hemoglobin estimation among the elderly. This will help us understand the burden and trend of anemia among the elderly and the effectiveness of any national program targeting elderly anemia.

Strength and limitations

This study bridges a knowledge gap by providing the first national-level statistics on the prevalence of anemia in the elderly in India. We assessed each study's risk of bias and conducted sub-group analyses to examine heterogeneity. We also utilized a conventional search method. Although we did not take into account studies that were written in languages other than English, we do not feel that this had an impact on our conclusions because, in India, practically all medical literature is published in English. As we know, an electronic search for observational studies identifies fewer than half of the studies, so other methods like a search of grey literature should have been employed. Though we pooled findings of several groups, such as urban, rural, community, and hospital, we performed sub-group analysis to explore the reason for heterogeneity. While we examined bibliographies extensively, it is likely that some information may not be identified by our searches. The pooled estimate of anemia emerging from this study needs to be interpreted along with the considerable heterogeneity observed between the studies. The studies included in the current meta-analysis also showed considerable publication bias. As there were a good number of studies, we conducted a sensitivity analysis. The observed prevalence of anemia, however, did not deviate significantly from the combined estimate derived from all the studies involved. Because of the significant variability between the studies, the pooled estimate of anemia that emerged from this analysis needs to be carefully interpreted. The limited number of studies conducted in rural settings that were included in this review restricts the generalizability of the findings because it is a crucial factor.

## Conclusions

As per the WHO criteria, the burden of anemia in the elderly age group is considerably high. The prevalence of anemia among the elderly in India was found to be even highest among all other age groups when compared with the NFHS-4 survey data. Considering the large heterogeneity between the studies, cautious interpretation has to be made about the results. Though there are some variations across geographical areas or urban-rural populations, the high prevalence was constant. The factors behind the causation, progression, and treatment outcomes need to be identified and addressed in order to achieve “active and healthy aging”.
